# Revisiting lung cancer immunotherapy in the era of long COVID: mechanistic insights and therapeutic implications

**DOI:** 10.3389/fcimb.2025.1657691

**Published:** 2025-11-04

**Authors:** Xingyao Lv, Lin Ji, Weijia Cao, Yanyan Xue, Hua Dai, Shengzhe Zhang

**Affiliations:** ^1^ Institute of Translational Medicine, School of Medicine, Yangzhou University, Yangzhou, Jiangsu, China; ^2^ The Key Laboratory of the Jiangsu Higher Education Institutions for Nucleic Acid & Cell Fate Regulation, Yangzhou University, Yangzhou, Jiangsu, China

**Keywords:** long covid, lung cancer, t-cell exhaustion, immune checkpoint inhibitors, pulmonary fibrosis, COVID-19 vaccination

## Abstract

In the post-COVID-19 era, understanding the long-term impact of Long COVID on the immune system is essential for deciphering its influence on lung cancer pathogenesis and immunotherapeutic efficacy. This review comprehensively examines how persistent COVID-19 sequelae-manifested as chronic inflammation, pulmonary fibrosis, cytokine dysregulation, and T-cell exhaustion can reshape the lung cancer microenvironment. In addition, the emerging roles of memory B cells and altered neutrophil function in promoting tumorigenesis are discussed. Importantly, we analyze recent clinical evidence suggesting that COVID-19 vaccination may enhance the efficacy of immune checkpoint inhibitors, potentially by modulating host immunity. By integrating mechanistic insights with clinical observations, this review aims to illuminate the challenges and opportunities at the intersection of Long COVID and lung cancer treatment, thereby fostering the development of personalized therapeutic strategies in the post-pandemic era.

## 1 Introduction

Severe acute respiratory syndrome coronavirus 2 (SARS-CoV-2), the causative agent of the COVID-19 pandemic emerging in late 2019, is a positive-sense, single-stranded RNA virus with a genome size of approximately 26–32 kb. Unlike retroviruses, the SARS-CoV-2 genome lacks a reverse transcriptase gene (Lu et al., 2020). Viral entry into host cells is primarily mediated by the interaction between the receptor-binding domain (RBD) within the S1 subunit of its spike (S) glycoprotein and the host cell receptor angiotensin-converting enzyme 2 (ACE2) (Zhang H. et al., 2020). Clinically, SARS-CoV-2 infection manifests with a broad spectrum of symptoms, ranging from fever, cough, and headache to fatigue, anosmia, and diarrhea (Li et al., 2023). While most individuals recover from the acute phase of infection, a substantial subset experiences a persistent constellation of debilitating symptoms collectively termed post-acute sequelae of SARS-CoV-2 infection (PASC), commonly known as “Long COVID.” Key features include profound fatigue, persistent respiratory difficulties, evidence of pulmonary fibrosis, and diverse neurological dysfunction (Roth et al., 2021). Critically, emerging evidence indicates that the immune dysregulation and chronic inflammation associated with Long COVID may exert downstream effects on tumorigenesis, potentially elevating cancer risk.

Concurrently, immune checkpoint inhibitors (ICIs) have revolutionized cancer immunotherapy, demonstrating significant improvements in survival outcomes for patients with lung cancer (Guibert and Mazières, 2015). Immune checkpoints are inhibitory surface receptors expressed on T cells and other immune cells, functioning as critical negative regulators of immune activation against various antigens, including tumor-associated antigens (Hodi et al., 2010). ICIs constitute a class of therapeutic monoclonal antibodies designed to block these inhibitory pathways, thereby removing barriers to T cell activation and harnessing the body’s intrinsic immune response against malignant cells (Liu and Sun, 2021). Among the most intensively studied pathways in lung cancer immunotherapy are the programmed death-1 (PD-1)/programmed death-ligand 1 (PD-L1) axis and the cytotoxic T-lymphocyte-associated protein 4 (CTLA-4) pathway (Cheng et al., 2024). Monoclonal antibodies targeting PD-1, CTLA-4, or PD-L1 have undergone extensive evaluation in advanced clinical trials. Substantial evidence confirms the promising clinical efficacy of ICI monotherapy and highlights the potential for synergistic effects in combination strategies for lung cancer treatment (Reckamp et al., 2022).

Recent research has increasingly focused on the profound and lasting impact of Long COVID on the host immune system. The immune response is broadly categorized into the innate and adaptive arms, which function interdependently. An effective defense against SARS-CoV-2 necessitates coordinated activity from both systems (Schultze and Aschenbrenner, 2021). The innate immune system includes granulocytes, monocytes, macrophages, natural killer (NK) cells, and dendritic cells, while the adaptive immune system relies on antigen-specific T cells and B cells (Sette and Crotty, 2021). A growing body of evidence indicates that Long COVID is characterized by significant immune alterations, prominently featuring T-cell exhaustion (Lin et al., 2020) alongside functional impairments in neutrophils (Pisareva et al., 2023) and B cells (Thieme et al., 2021). Notably, in severe acute COVID-19, dysregulated immune activation can culminate in a life-threatening “cytokine storm” (Passaro et al., 2021). In the post-COVID-19 era, understanding the specific and long-term consequences of Long COVID on the host’s immune system is paramount, particularly for lung cancer patients receiving ICIs, whose treatment efficacy hinges on a functional immune system.

This review explores the emerging intersection between Long COVID and lung cancer, framing a novel perspective on how the persistent immune dysregulation following SARS-CoV-2 infection could contribute to long-term oncogenesis. Specifically, we examine how immunological changes such as T cell exhaustion, cytokine dysregulation, and pulmonary fibrosis in Long COVID may affect the tumor microenvironment and immunotherapeutic efficacy in lung cancer. By synthesizing existing mechanistic studies and experimental data, this report elucidates the potential mechanisms of interaction between the altered immune system in Long COVID and lung cancer immunotherapy, providing a basis for future clinical and research strategies.

## 2 The association between long COVID and the incidence of lung cancer

Accumulating epidemiological evidence points to a significant association between SARS-CoV-2 infection and an elevated risk of lung cancer development, particularly among individuals experiencing persistent symptoms following prolonged COVID-19 infection. This potential link has garnered increasing attention within the medical research community (Huang et al., 2021). Notably, cancer patients themselves exhibit heightened susceptibility to SARS-CoV-2 infection and experience poorer clinical outcomes. This susceptibility and prognostic significance are primarily reflected in increased mortality risk (Oldani et al., 2022), more severe clinical manifestations (Dai et al., 2020), and a higher likelihood of infection (Wang et al., 2021). This section delineates the postulated mechanisms underpinning the potential association between Long COVID and lung carcinogenesis.

### 2.1 Post-infection pulmonary complications and fibrosis

**Figure 1:**
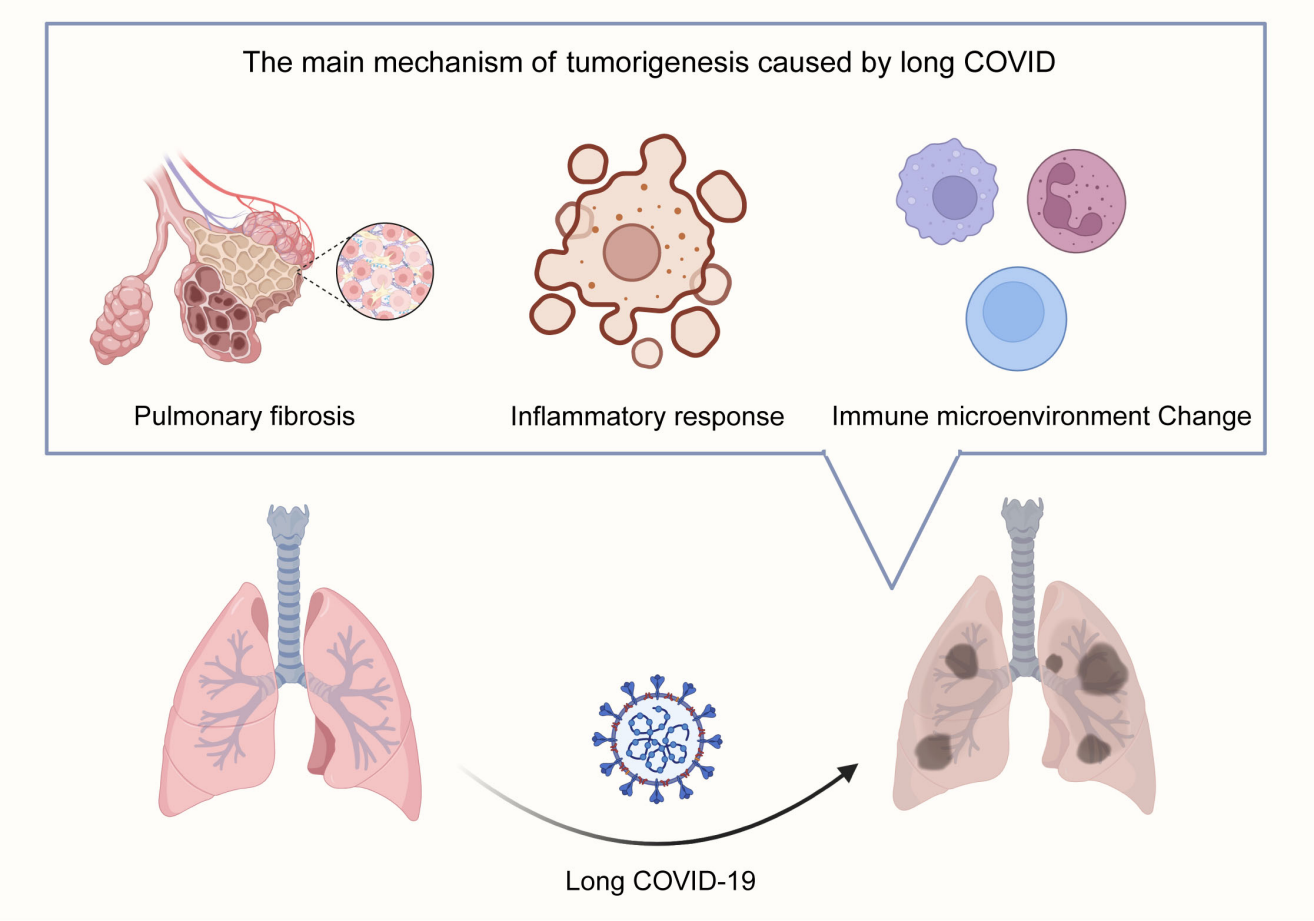
Proposed Mechanisms Linking Long COVID to Lung Carcinogenesis. Long COVID promotes lung tumorigenesis through three interconnected pathways: 1. Pulmonary Fibrosis: Persistent interstitial abnormalities and fibrotic scar formation post-SARS-CoV-2 infection create a pro-tumorigenic microenvironment, mirroring idiopathic pulmonary fibrosis-associated lung cancer risk. 2. Chronic Inflammation & Cytokine Dysregulation: Sustained elevation of pro-inflammatory cytokines (e.g., IL-6, TNF-α) via IL-6 amplifier and NF-κB pathways drives cellular transformation and tumor promotion. 3. RAAS Dysregulation: SARS-CoV-2-mediated ACE2 downregulation disrupts angiotensin balance, favoring the pro-fibrotic/pro-inflammatory Ang-II/AT1R axis over the protective Ang-(1-7)/MasR axis, further supporting tumor growth.

Substantial evidence indicates that SARS-CoV-2 infection can lead to long-term pulmonary complications (Das et al., 2017). Longitudinal studies tracking recovered patients for up to 15 years reveal chronic lung involvement characterized by persistent interstitial abnormalities detectable years post-infection (Zhang P. et al., 2020). Imaging analyses further demonstrate that a significant proportion (approximately one-third) of recovered patients exhibit radiological signs suggestive of pulmonary fibrosis (Das et al., 2017). Recently, several researchers proposed that pulmonary fibrosis may be a potential long-term complication associated with COVID-19 (Spagnolo et al., 2020). Critically, a well-established body of research directly links pulmonary fibrosis with an increased risk of lung cancer, with reported prevalence rates ranging from 2.7% to 48% among patients with idiopathic pulmonary fibrosis (Ballester et al., 2019). The fibrotic microenvironment induced by Long COVID may thus provide a fertile ground for lung cancer initiation and progression (**Figure 1**).

### 2.2 Chronic inflammation and cytokine dysregulation

Chronic inflammation is a widely recognized critical driver of carcinogenesis across multiple cancer types (Grivennikov et al., 2010; O’Callaghan et al., 2010), significantly contributing to tumor initiation, promotion, and progression (Atsumi et al., 2014; Munn, 2017). In the context of Long COVID, the pulmonary milieu is often characterized by a sustained inflammatory state. This persistent inflammatory microenvironment may foster conditions conducive to lung carcinogenesis. Key inflammatory pathways implicated include the IL-6 amplifier mechanism, involving STAT3 activation by IL-6 and NF-κB activation by IL-17 or TNF-α (Hirano, 2021). These cytokines are known to play significant roles in tumor promotion and cellular transformation within lung cancer pathogenesis (Block et al., 2012; Kiuchi et al., 1999). SARS-CoV-2 infection can trigger a profound release of pro-inflammatory cytokines, often termed a “cytokine storm,” with IL-6 frequently identified as a central mediator (Coomes and Haghbayan, 2020; Lokau et al., 2024; Yang et al., 2021). Furthermore, the clinical relevance of this axis is underscored by studies demonstrating reduced mortality in critically ill COVID-19 patients treated with IL-6 receptor antagonists, highlighting the pivotal role of cytokine dysregulation in disease severity and potentially long-term sequelae (Fuller and Chagla, 2023; Masiá et al., 2022; Zhou and Price, 2020) (**Figure 1**).

### 2.3 Dysregulation of the renin-angiotensin-aldosterone system

The renin-angiotensin-aldosterone system (RAAS) is crucial for chronic blood pressure and vascular resistance regulation. Components of this system, particularly angiotensin-converting enzyme 2 (ACE2), angiotensin (1-7) [Ang (1-7)], and angiotensin II (Ang-II), have been increasingly implicated in the progression of various malignancies, including from early stages (Fountain et al., 2025). Ang-II, a key effector peptide, has been associated with carcinogenesis, metastasis, and recurrence, potentially through mechanisms involving cancer stem cell formation (Deshayes and Nahmias, 2005). In non-small cell lung cancer (NSCLC), Ang-II modulates cancer cell invasiveness and cancer stem cell populations (Tawinwung et al., 2015). Furthermore, high ACE2 expression has been proven to be correlated with malignancy and poor prognosis in certain cancers (Xu et al., 2017). SARS-CoV-2 utilizes ACE2 as its primary cellular receptor for entry. Infection leads to ACE2 downregulation, disrupting the balance between the pro-inflammatory/pro-fibrotic Ang-II/AT1R axis and the counter-regulatory Ang (1-7)/MasR axis. This RAAS dysregulation, a hallmark of both severe COVID-19 and Long COVID, may represent another pathway linking persistent infection to an altered lung microenvironment favoring tumorigenesis (Feng et al., 2010; Sommerstein et al., 2020) (**Figure 1**).

## 3 Shared mechanistic pathways linking long COVID and lung cancer

### 3.1 Chronic inflammation and cytokine storm

Persistent inflammation, characterized by elevated levels of pro-inflammatory cytokines (IL-6, TNF-α, IL-1β), is a hallmark of Long COVID (Coomes and Haghbayan, 2020; Lokau et al., 2024). Similarly, chronic inflammation is a well-established driver of lung cancer development and progression (Coussens and Werb, 2002). In Long COVID, sustained cytokine production can lead to immune cell dysfunction, tissue damage, and altered cellular signaling, potentially creating a permissive environment for malignant transformation or promoting the growth of pre-existing cancerous cells. Quantitatively, studies have shown that patients with severe Long COVID exhibit a 2–3 fold increase in IL-6 levels compared to recovered individuals without persistent symptoms (Lokau et al., 2024).

### 3.2 Immune dysregulation and T cell exhaustion

Both Long COVID and cancer are associated with profound alterations in immune cell function, including T cell exhaustion, increased expression of immune checkpoint molecules (PD-1, CTLA-4), and impaired antigen presentation (Barber et al., 2006; Rha and Shin, 2021). In Long COVID, chronic antigen stimulation and inflammation can drive T cell exhaustion, reducing their ability to effectively eliminate infected or transformed cells. This immune dysfunction can also impair the efficacy of cancer immunotherapies. For example, PD-1 expression on CD8^+^ T cells is significantly higher in Long COVID patients compared to healthy controls (p<0.05) (Silva et al., 2023).

### 3.3 Tissue remodeling and fibrosis

Pulmonary fibrosis is a significant concern in Long COVID patients, with some studies reporting fibrosis in up to 38% of severe cases (Babar et al., 2024). Fibrosis alters the lung architecture, disrupts immune cell trafficking, and promotes the release of pro-tumorigenic factors, such as TGF-β (Wynn et al., 2008). The altered extracellular matrix can also hinder drug delivery and reduce the effectiveness of immunotherapies.

## 4 Immunopathology of long COVID: reshaping the lung cancer microenvironment

**Figure 2:**
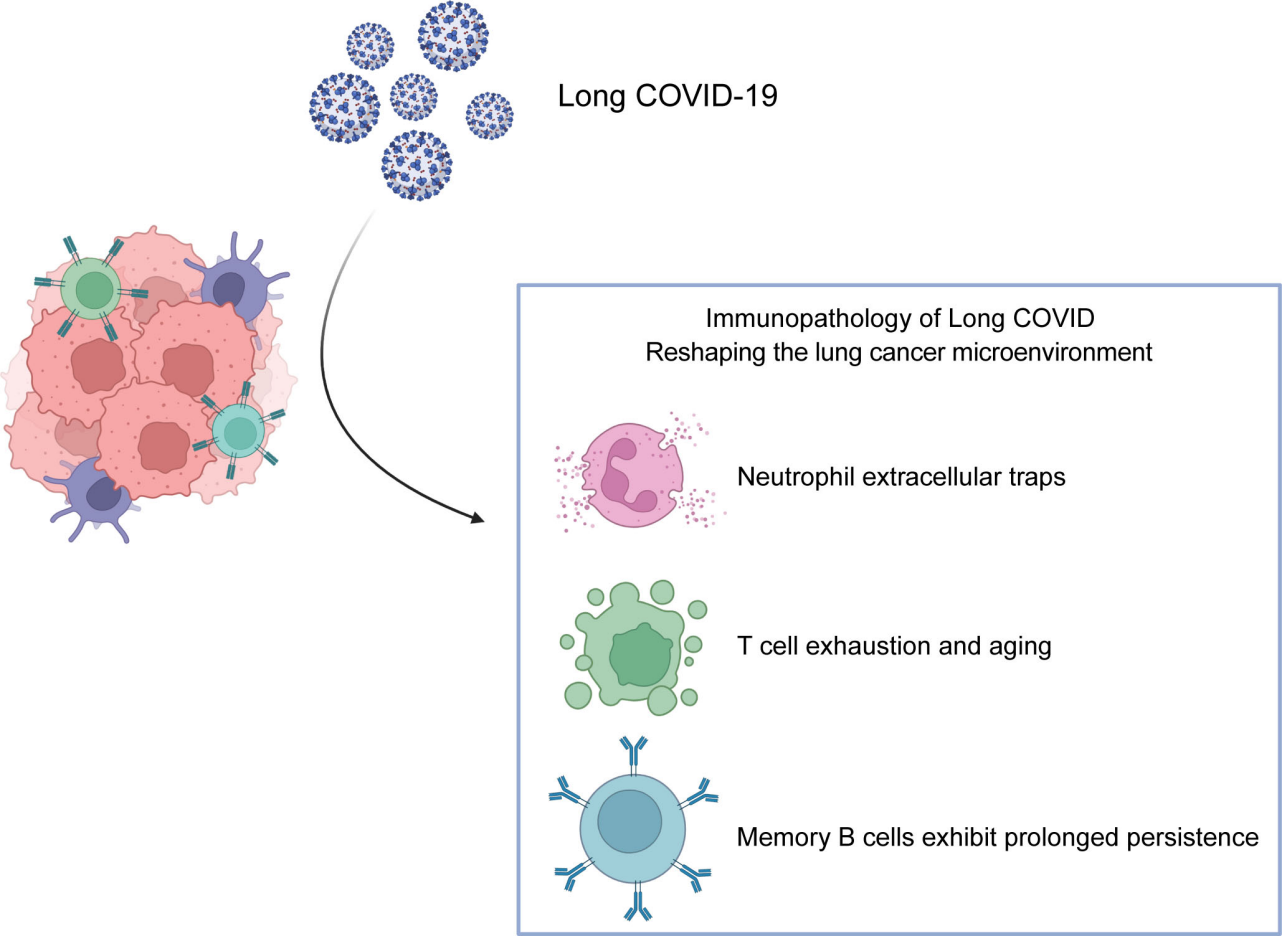
Immunopathological Features of Long COVID Reshaping the Lung Cancer Microenvironment. Key immune alterations in Long COVID remodel the lung tumor immune landscape: 1. T Cell Exhaustion and Senescence: Chronic antigen exposure drives CD8^+^ T cell dysfunction, characterized by upregulated inhibitory receptors (PD-1, CTLA-4), reduced proliferative capacity, and impaired effector function, dampening anti-tumor immunity. 2. Persistence of SARS-CoV-2-Specific Memory B Cells: Long-lived Memory B cells generated post-infection may influence tumor progression through antibody-dependent mechanisms or modulation of T cell responses, though their exact role in lung cancer immunotherapy remains unclear. 3. Neutrophil Extracellular Traps (NETs) and Dysregulated Activity: Enhanced NETosis (releasing DNA/MPO/NE complexes) and elevated neutrophilic chemokines (e.g., CXCL8) sustain chronic inflammation and tissue damage, polarizing neutrophils toward pro-tumorigenic (N2) phenotypes in the tumor microenvironment.

Long COVID is associated with profound and persistent alterations in immune cell composition and function. Studies report significant increases in neutrophils, monocytes, NK cells, and CD4^+^ T cells in Long COVID patients, alongside decreases in total lymphocytes and CD8^+^ T cells. Cytokine profiling reveals significantly elevated levels of IL-6, TNF-α, IFN-γ, IL-2, IL-4, and IL-10 in hospitalized patients, suggesting potential skewing of CD4^+^ T cell differentiation (Lin et al., 2020). This section explores how key immunological features of Long COVID-T cell exhaustion, persistent memory B cell responses, and neutrophil extracellular trap (NET) formation – may impact the lung cancer immune microenvironment (summarized in **Figure 2** and **Table 1**).

**Table 1:** Immunological alterations in long COVID relevant to lung cancer microenvironment.

Immune alteration	Key findings in long COVID	References
T cell Dysfunction	Significant reduction in adaptive immune cells (T cells, particularly RTEs, and B cells) at 10 months post-infection.	(Kratzer et al., 2024)
	Diminished CD8^+^ T cell response relative to CD4^+^ T cells at 6 months post-infection.	(Kumar et al., 2021)
	PD-1^+^ SARS-CoV-2-specific CD8^+^ T cells may retain functionality.	(Rha et al., 2021)
Memory B Cell Persistence	Persistent Spike IgG titers, stabilizing around 6 months post-infection.	(Achiron et al., 2021)
	Long-term persistence of SARS-CoV-2-specific memory B cells.	(Cohen et al., 2021)
Neutrophil Dysregulation & NETs	Persistently elevated serum NET markers (NE, MPO, cfDNA) for ≥6 months post-infection.	(Pisareva et al., 2023)
	Upregulation of neutrophil activation signatures and inflammatory chemokines in airways >10 months post-infection.	(Gerayeli et al., 2024)
	Elevated neutrophilic chemokines (CXCL8), inflammasome pathway components (NLRP3, IL-18/IL-18R1) correlating with persistent ILD.	(George et al., 2022)

RTE, (Recent Thymic Emigrant); NETs, (Neutrophil Extracellular Traps); NE, (Neutrophil Elastase); MPO, (Myeloperoxidase); cfDNA, (Cell-free DNA); ILD, (Interstitial Lung Disease).

### 4.1 T cell exhaustion and dysfunction

T cells are central orchestrators of adaptive immunity, critical for maintaining health and combating disease. T cell development occurs in the thymus. Following antigen encounter in acute settings, naïve T cells differentiate into effector and memory subsets, which mediate direct killing, diversified immune regulatory functions and long-term protection. However, chronic antigen exposure, as seen in persistent viral infections and cancer, can drive T cells towards a state of functional exhaustion, characterized by progressive loss of effector function and sustained expression of inhibitory receptors like PD-1 and CTLA-4 (Sun et al., 2023). This shared pathway of exhaustion underpins the conceptual overlap between immunotherapy strategies for chronic infections and cancer.

While much research focused on the acute phase of COVID-19, understanding the longitudinal immune trajectory in Long COVID is paramount. T cells are pivotal mediators of the host response to SARS-CoV-2. Longitudinal studies examined patients recovering from mild, moderate, and severe COVID-19 at two time points (3- and 6-months post-infection), focusing on the dynamic changes in T cell profiles post-infection. Overall, a trend towards exhaustion is observed, particularly in CD8^+^ T cells. Dysregulated immunity in CD4^+^, CD8^+^, and Treg subsets often persists for the first 3 months, with a partial functional shift becoming apparent between 3–6 months, the extent of which varies with initial infection severity. This manifests itself as long-lasting attenuated T cell activation, reduced proliferative capacity, and a shift towards an exhausted/senescent phenotype, particularly within the CD8^+^ compartment. Together with prolonged unresolved inflammation, this T cell dysfunctions likely contributes to compromised anti-tumor immunity (Wiech et al., 2022).

Paradoxically, some studies report that PD-1-expressing SARS-CoV-2-specific CD8^+^ T cells retain functionality rather than being fully exhausted (Rha et al., 2021). This complexity suggests that severe outcomes in COVID-19, including Long COVID complications and potentially increased cancer susceptibility, may stem from a combination of SARS-CoV-2 infection, potential co-pathogens, and the resultant induction of immune cell dysfunction, prominently featuring T cell exhaustion (Roe, 2021).

### 4.2 Persistence of SARS-CoV-2-specific memory B cells

B cells, originating from bone marrow hematopoietic stem cells, undergo maturation and selection before populating peripheral lymphoid organs (Nemazee, 2017). Antigen-driven B cell responses involve classical germinal center (GC) reactions, leading to affinity maturation and long-lived plasma/memory cells, and extrafollicular (EF) responses (Victora and Nussenzweig, 2022). While some infections/vaccines confer long-lasting, sometimes lifelong robust immunity, immunity against respiratory viruses like coronaviruses tend to be less durable (Morens et al., 2023). In the “suppressed” immune microenvironment of chronic conditions, including Long COVID and cancer, activating B cells presents a potential therapeutic avenue. Analogous to chronic infections, tumor-infiltrating B lymphocytes (TIL-Bs) can exert anti-tumor effects via antibody production, T cell modulation, and direct cytotoxicity (Wang et al., 2019). It remains to be determined whether the specific memory B cells generated by the host in response to SARS-CoV-2 infection influence lung cancer progression or response to therapy, or whether strategies modulating these cells impact immunotherapy efficacy. Evidence from HPV-associated head and neck cancer suggests antigen-specific B cells may represent a novel approach for tumor immunotherapy (Wieland et al., 2021). Adaptive immunity to SARS-CoV-2, comprising antibodies, memory B cells, and T cells, protects against reinfection (Sariol and Perlman, 2020). Serum antibody levels typically peak around 2–3 weeks post-infection and may wane over time (Dan et al., 2021; Marot et al., 2021).

Among the majority of people, anti-SARS-CoV-2 serum antibodies persist for more than six months after the initial infection, but some patients lose their specific antibodies rapidly (Seow et al., 2020; Zheng et al., 2021). Spike-specific IgG titers often persist for months, stabilizing at a protective plateau around 6 months and remaining detectable for at least 9 months in many convalescents (Achiron et al., 2021). Crucially, memory B cells exhibit remarkable longevity. They can persist for years, poised to rapidly differentiate into antibody-secreting plasma cells upon re-exposure (Stamper et al., 2020). Numerous studies confirm the persistence of SARS-CoV-2-specific memory B cells long after acute infection, making them a reliable correlate of durable humoral immunity (Cohen et al., 2021; Pušnik et al., 2021; Winklmeier et al., 2022). Consequently, quantifying these cells provides a robust indicator of long-term immune memory in Long COVID (Thieme et al., 2021).

### 4.3 Neutrophil extracellular traps and dysregulated neutrophil activity

Neutrophils, a subset of myeloid white blood cells, serve as primary responders to acute inflammatory and infection. In humans, neutrophils constitute 50-70% of the circulating leukocyte population (Doeing et al., 2003; Mestas and Hughes, 2004). As first responders, they combat pathogens via phagocytosis, degranulation, cytokine production, and the formation of neutrophil extracellular traps (NETs) – webs of DNA decorated with antimicrobial proteins (Mantovani et al., 2011; Papayannopoulos, 2018). During the initial phase of COVID-19 infection, neutrophils are recruited to the lungs where they eliminate the invading SARS-CoV-2 via multiple mechanisms. While essential for host defense, aberrant neutrophil activation is a hallmark of severe COVID-19, contributing to cytokine storms and exaggerated host immune responses in COVID-19 patients (Barnes et al., 2020). Persistent neutrophil infiltration signifies chronic inflammation and can drive tissue damage.

Within the tumor microenvironment (TME), neutrophils exhibit functional plasticity. They are often categorized as anti-tumor (N1 neutrophils) or pro-tumor (N2 neutrophils) phenotypes (Fridlender et al., 2009). Tumor-derived factors, particularly the immunosuppressive cytokine TGF-β, promote polarization towards the pro-tumorigenic N2 state (Pylaeva et al., 2016). Neutrophil depletion studies in mice show modest anti-tumor effects, while TGF-β blockade via the TGF-β receptor inhibitor SM16 promotes accumulation of anti-tumor N1 neutrophils; subsequent neutrophil depletion under these conditions then accelerates tumor growth, highlighting their context-dependent role (Patel et al., 2018). Additionally, type I interferons appear involved in inducing N1 polarization post-TGF-β inhibition (Andzinski et al., 2016). In chronic inflammation, distinct neutrophil subsets recruit different immune infiltrates, exerting divergent effects on tumors. This evidence suggests that targeting neutrophils polarization or NETosis thus represents a promising therapeutic strategy for both COVID-19 sequelae and lung cancer.

Substantial evidence indicates dysregulated neutrophil activity in Long COVID. Circulating neutrophils from Long COVID patients demonstrate heightened NET formation (George et al., 2022; Krinsky et al., 2023; Woodruff et al., 2023). Longitudinal monitoring of NET markers (serum neutrophil elastase (NE), myeloperoxidase (MPO), cell-free DNA) in previously hospitalized patients shows levels remain significantly elevated for at least 6 months post-infection compared to controls, albeit lower than during acute infection (Pisareva et al., 2023). In the context of Long COVID, compared to age and gender-matched healthy controls, survivors also exhibit significantly higher levels of detectable antinuclear antibodies at both 3- and 12-months post-infection (Son et al., 2023). Patients with persistent post-COVID interstitial lung abnormalities show elevated neutrophil counts and serum MPO, correlating with radiological disease extent. Proteomic analysis identifies the neutrophil chemotactic factor IL-17 and neutrophil chemokines (CXCL1, CXCL8) as significantly associated with persistent lung disease and functional impairment (George et al., 2022). Furthermore, single-cell transcriptomic analysis of airways in Long COVID patients (>10 months post-acute infection) reveals increased neutrophil abundance and upregulated neutrophil activation signatures alongside inflammatory chemokines across multiple cell clusters, pointing to sustained neutrophilic inflammation (Gerayeli et al., 2024).

## 5 Lung cancer immunotherapy in the context of COVID-19 vaccination

Immune checkpoints (ICs) like PD-1, CTLA-4, and TIM-3, expressed on various immune cells (T cells, NK cells, DCs), deliver inhibitory signals that suppress immune activation. In chronic conditions like cancer and persistent infections, sustained IC/ligand expression drives T cell exhaustion, enabling antigen-mediated immune escape. ICIs, which block these pathways, have demonstrated significant efficacy in treating various cancers (Dyck and Mills, 2017; Wykes and Lewin, 2018). Although many studies have revealed that ICI immunotherapy holds promising clinical prospects in patients with infectious diseases (Achiron et al., 2021), key questions arise regarding their interaction with SARS-CoV-2 infection and vaccination in lung cancer patients: Does ICI reduce SARS-CoV-2 viral load? Should ICI regimens be adjusted upon SARS-CoV-2 infection? This section focuses on the potential interactions between ICI therapy for lung cancer and COVID-19 vaccination.

### 5.1 Safety of ICI during the COVID-19 pandemic: a nuanced picture

The association between ICI treatment for lung cancer and COVID-19 severity remain debated. Some studies suggest older age and ICI treatment correlate with worse COVID-19 outcomes (Robilotti et al., 2020; Zhou et al., 2020). Conversely, other studies including larger cohorts, report no significant association between ICI monotherapy and increased COVID-19 severity or mortality (Grivas et al., 2021; Rogiers et al., 2021). Most evidence indicates that ICI immunotherapy alone is not strongly associated with heightened COVID-19 severity. A clinical study of 69 lung cancer patients with COVID-19 (41 previously treated with PD-1 blockade, 28 untreated) found no difference in COVID-19 severity between groups (Luo et al., 2020). Furthermore, a large multicenter observational study (TERAVOLT) indicated that various systemic anti-cancer therapies (including pembrolizumab, nivolumab, ipilimumab, ICI-chemotherapy combinations, TKIs, chemotherapy alone) did not significantly affect the survival rate in lung cancer patients with COVID-19 (Garassino et al., 2020).

However, an important caveat emerges regarding combination ICI therapy. Lung cancer patients treated with dual immunotherapy (e.g., nivolumab + ipilimumab) may face a higher risk of severe complications or death if infected with SARS-CoV-2. A 70-year-old patient diagnosed with COVID-19 and lung adenocarcinoma received the dual immunotherapy (nivolumab and ipilimumab) but died from cytokine release syndrome (Murata et al., 2022). Additionally, clinical cases report fatalities due to cytokine release syndrome (CRS) in such patients, and registry data indicate significantly higher mortality rates associated with combination ICI compared to monotherapy (Owonikoko et al., 2021).

Intriguingly, emerging data suggest potential immunostimulatory effects. Compared to the general population, NSCLC patients receiving pembrolizumab combined with chemotherapy exhibited stronger humoral immune responses to SARS-CoV-2 infection (higher SARS-CoV-2 reactive IgG, neutralizing antibodies) and enhanced cellular immunity (sustained increases in follicular helper T cells, activated CD4^+^ and CD8^+^ T cells) (Mellinghoff et al., 2021). This hints at a potential “mutually beneficial” interaction between certain anti-cancer immunotherapies and anti-viral immunity.

### 5.2 Exploring a “mutually beneficial” interaction

Clinical observations suggest that cancer patients receiving ICI may have a lower risk of SARS-CoV-2 infection or experience milder COVID-19 symptoms (Moritz et al., 2021). The mechanistic basis for this phenomenon likely relates to the restoration of T cell function by ICI. Preclinical evidence indicates that anti-PD-1 therapy can reinvigorate exhausted antiviral T cell responses, reducing viral load (Barber et al., 2006). Supporting this, clinical cases describe lung cancer patients receiving ICI who contracted COVID-19 did not exhibit obvious signs of pulmonary involvement (Yang and Xu, 2023).

Conversely, COVID-19 vaccination may enhance the efficacy of ICI in cancer treatment. Global vaccination efforts have deployed various platforms, including mRNA-based vaccines and adenovirus vector vaccines expressing the SARS-CoV-2 spike protein (CoV-2-S). Specifically, nucleoside-modified mRNA encoding antigens encapsulated in LNP (mRNA-LNP) have shown great promise and been widely accepted, demonstrating an efficacy of approximately 95% in healthy subjects (Baden et al., 2021; Polack et al., 2020). Recent research explores vaccines designed to elicit robust CD8^+^ cytotoxic T lymphocyte (CTL) responses against SARS-CoV-2, particularly relevant for high-risk groups like cancer patients. Preclinical studies demonstrate that vaccines inducing lung-homing T cells or dual-antigen vaccines targeting both tumor-associated antigens (TAAs) and SARS-CoV-2 can generate potent anti-tumor effects alongside virus-specific CTLs (Shimizu et al., 2022).

Critically, clinical retrospective evidence supports this synergy. A retrospective study analyzed the survival data of 104 stage III-IV NSCLC patients treated with ICI. Compared with the unvaccinated group, the Overall Response Rate (ORR: 28.0% vs. 11.39%, p = 0.05) was significantly improved in the COVID-19 vaccinated group. Regarding long-term survival benefits, the COVID-19 vaccine had a profound impact on the progression free survival (PFS: HR = 0.16, p = 0.021) and overall survival (OS: HR = 0.168, p = 0.019) of NSCLC patients treated with ICI. Compared with the unvaccinated group, Both PFS (p < 0.001) and OS (p < 0.001) were significantly prolonged in the vaccinated group. Furthermore, vaccinated patients exhibited higher circulating CD4^+^ T cell levels (p = 0.047). This study strongly suggests that COVID-19 vaccination enhances the efficacy of anti-PD-1 immunotherapy in advanced NSCLC, potentially offering additional survival benefits for these NSCLC patients (Qian et al., 2023).

## 6 Discussion and conclusion

This review underscores the critical need to understand the impact of Long COVID on the host immune system, particularly concerning lung cancer pathogenesis and immunotherapy efficacy. Long COVID, characterized by persistent symptoms and profound immune alterations-including T cell exhaustion, dysregulated neutrophil activity, and persistent but altered B cell memory-can significantly reshape the tumor immune microenvironment. These changes hold substantial implications for the effectiveness of immunotherapies like ICI, which are crucial for lung cancer treatment.

We have outlined the potential mechanisms by which Long COVID may contribute to lung cancer development, encompassing post-infection pulmonary fibrosis, chronic inflammation, cytokine imbalance, and dysregulation of the RAAS. Furthermore, we explored how Long COVID-associated immunopathology, notably involving dysfunctional T cells, persistent memory B cells, and neutrophil extracellular traps, may remodel the lung cancer microenvironment. The evolving landscape also reveals complex interactions between lung cancer immunotherapy and COVID-19 vaccination. While the safety of ICI monotherapy during the pandemic appears generally acceptable, caution is warranted with combination ICI regimens. Importantly, emerging clinical evidence points towards a potential “mutually beneficial” relationship, where ICI might modulate anti-viral responses and COVID-19 vaccination could enhance ICI efficacy in lung cancer patients, significantly improving survival outcomes.

In conclusion, the immunologic perturbations and tissue remodeling associated with Long COVID present distinct challenges for lung cancer patients undergoing immunotherapy. Moving forward, research efforts should prioritize:

Investigating the Molecular Mechanisms: Elucidating the specific molecular mechanisms by which Long COVID-related immune dysregulation (e.g., persistent cytokine signaling, altered T cell receptor repertoire) promotes lung cancer development and progression. This includes identifying novel therapeutic targets to interrupt these pathways.

Conducting Longitudinal Studies: Implementing large-scale longitudinal studies to assess the long-term cancer risk in Long COVID patients, with comprehensive immune profiling and clinical follow-up.

Developing Targeted Therapeutic Strategies: Designing and testing targeted therapeutic strategies to mitigate the pro-tumorigenic effects of Long COVID, such as combining anti-inflammatory agents with immunotherapies or developing novel interventions to restore T cell function and reverse fibrosis.

Applying Multi-omics Approaches: Utilizing multi-omics approaches (genomics, proteomics, metabolomics) to identify predictive biomarkers for cancer risk and immunotherapy response in Long COVID patients.
